# Public health and clinical implications of *Dobbs v*. *Jackson* for patients and healthcare providers: A scoping review

**DOI:** 10.1371/journal.pone.0288947

**Published:** 2024-03-29

**Authors:** David T. Zhu, Lucy Zhao, Tala Alzoubi, Novera Shenin, Teerkasha Baskaran, Julia Tikhonov, Catherine Wang

**Affiliations:** 1 Medical Scientist Training Program, School of Medicine, Virginia Commonwealth University, Richmond, VA, United States of America; 2 Faculty of Health Sciences, McMaster University, Hamilton, ON, Canada; 3 Faculty of Health Sciences, University of Waterloo, Waterloo, ON, Canada; 4 Bayview Secondary School, Richmond Hill, ON, Canada; 5 School of Interdisciplinary Sciences, Faculty of Science, McMaster University, Hamilton, ON, Canada; 6 Faculty of Medicine and Health Sciences, McGill University, Montreal, QC, Canada; University of Foggia: Universita degli Studi di Foggia, ITALY

## Abstract

**Introduction:**

On June 24, 2022, the U.S. Supreme Court’s decision in *Dobbs v*. *Jackson* reversed the precedent set forth by *Roe v*. *Wade*, empowering individual states to regulate abortion care. This aftermath of this ruling has given rise to widespread bans, limiting the accessibility of abortion services for patients and impeding providers’ ability to deliver a comprehensive spectrum of reproductive health services. Of particular concern is the disproportionate impact on medically underserved groups, further heightening existing social and structural disparities in reproductive health.

**Methods:**

We conducted a scoping review to broadly evaluate the clinical and public health impact of *Dobbs* on patients’ access to abortion care and related reproductive health services, in addition to the training and clinical practice of healthcare providers. We searched eight bibliographic databases (PubMed, Scopus, Embase, PsycINFO, Google Scholar, Science Direct, JSTOR, and Web of Science) and three preprint servers (medRxiv, bioRxiv, and Europe PMC) using various combinations of keywords related to ‘abortion’, ‘Dobbs’, and ‘Roe’ on March 22, 2023. Four reviewers independently screened the studies based on pre-specified eligibility criteria and one reviewer performed data extraction for pre-identified themes. The search was conducted based on PRISMA Extension for Scoping Reviews (PRSIMA-ScR) guidelines.

**Results:**

Eighteen studies, comprising 12 peer-reviewed articles and 6 study abstracts, met the inclusion criteria. The studies demonstrated that *Dobbs* increased demand for contraception, magnified existing travel- and cost-related barriers to access, further polarized views on abortion and complex family planning on social media (e.g., Twitter), and evoked substantial concerns among medical trainees regarding their scope of practice and potential legal repercussions for providing abortion care.

**Conclusion:**

In the wake of *Dobbs v*. *Jackson*, further public health and clinical interventions are urgently needed to bridge disparities in abortion care and reproductive health, mitigating the deleterious consequences of this emerging public health crisis.

## 1. Introduction

The U.S. Supreme Court’s decision in *Dobbs v*. *Jackson Women’s Health Organization* in June 2022 reversed the longstanding federal protection for abortion care in the U.S., initially set forth by *Roe v*. *Wade* in 1973 [1, 2]. Although safe and effective, the medical provision of abortions has long stood as a contentious socio-political and religious policy issue in the U.S., including the Hyde Amendment, global gag rule, and many others [2]. *Dobbs* grants individual states varying degrees of authority to regulate and restrict access to abortions, with some states—particularly in Southern regions of the U.S.—being disproportionately affected by restrictions [1, 2]. Currently, more than half of U.S. states have implemented partial or complete bans on induced abortions [2, 3].

Approximately 60% of women of reproductive age live in U.S. states that are “hostile” to abortions [3]. A recent modeling study predicts that a nationwide abortion ban would increase maternal mortality from childbirth or pregnancy complications by 21% in the general U.S. population and 33% among Black Americans, exacerbating existing structural disparities in reproductive health for which Black individuals capable of pregnancy have traditionally faced greater barriers [4]. Other traditionally marginalized or medically underserved populations that have limited access to primary care or obstetrics and gynecology (OB-GYN) providers—including patients from low-income, Hispanic/Latinx, or rural backgrounds—are likely to also experience disproportionately worse access to abortion care and reproductive health services [5, 6].

Global epidemiological evidence indicates that laws restricting access to abortion care does not reduce the overall frequency of abortions; instead, it merely limits the rate of legal abortions while increasing the rate of unsafe abortions, linked to higher health risks and long-term complications [7, 8]. The denial of abortion services, as highlighted by The Turnaway Study, has significant public health consequences, including an increased risk of maternal morbidity and mortality, complications from unsafe clandestine abortions, psychological distress, financial strain, deleterious impacts on relationships, and more [9]. *Dobbs* is likely to worsen existing barriers, particularly affecting financial and geographical access. For instance, among those living in abortion-hostile states will likely need to travel out-of-state to access abortion or reproductive health services, many low-income patient populations will be systematically denied these opportunities [10, 11]. A recent commentary highlights that, under the *Dobbs* ruling, women who lack the ability to terminate a pregnancy against their wishes are likely to experience an increased risk of intimate partner violence (IPV) [12]. Further, the long-term consequences of *Dobbs* may potentially have indirect or spillover effects on other fields tangential to obstetrics, such as healthcare provision for congenital diseases and neonates [13], although further research is needed.

Finally, the *Dobbs* ruling is also likely to affect healthcare providers and trainees providing abortion care and reproductive health services. Anticipated challenges for healthcare professionals may include navigating the uncertainty of rapidly evolving legal restrictions on abortion care, the fear of prosecution or potential loss of medical licenses, facing stigma in the medical field, and more [14, 15]. Moreover, the potential conflicts between healthcare providers’ personal beliefs and their professional responsibilities surrounding issues such as bodily autonomy and reproductive rights may increase the risk of emotional distress, moral injury, and burnout [14, 15]. As such, the *Dobbs* ruling may impose an enduring threat to the well-being of the healthcare workforce across the U.S., particularly in OB-GYN and related fields.

To the best of our knowledge, no previous reviews have comprehensively examined the public health and clinical implications of *Dobbs* on patients and providers. To address this gap in the literature, we conducted a scoping review to overview the impact of *Dobbs* on (1) patients’ health outcomes and access to abortion care, and (2) medical trainees’ access to abortion training; and (3) providers’ ability to provide the full spectrum of reproductive health services.

## 2. Methods

### 2.1 Methodological approach

We conducted a scoping review in accordance with the methodological framework created by Arksey & O’Malley [16] and the PRISMA extension for Scoping Reviews (PRISMA-ScR) guidelines [17] **(S1 Table)** to capture both peer-reviewed and study abstracts related to the public health and clinical implications of *Dobbs v*. *Jackson*. Given the novelty and rapidly evolving nature of this topic, the flexibility and breadth of a scoping review design is well-suited to address our research objectives.

### 2.2 Information sources and search strategy

We conducted a literature search in eight bibliographic databases (PubMed, Scopus, Embase, PsycINFO, Google Scholar, Science Direct, JSTOR, and Web of Science) to capture published peer-reviewed studies, in addition to three other servers (medRxiv, bioRxiv, and Europe PMC) to capture preprint studies. Various combinations of the search terms ‘Dobbs’, ‘Roe’, ‘abortion’, ‘pregnancy termination’, ‘unintended pregnancy’, ‘abortifacient’, ‘misopristol’, ‘mifeprex’, ‘mifepristone’, ‘cytotec’, were used to retrieve articles on March 22, 2023. Since the *Dobbs v*. *Jackson* ruling occurred in June 2022, we optimized our search by restricting the date of publication from 2022 to 2023. The detailed search strategy, including combinations of MeSH terms and Boolean operators, can be found in **(S1 Table)**.

### 2.3 Selection of sources of evidence

Four reviewers (LZ, TA, NS, TB) independently screened study titles and abstracts using pre-specified inclusion and exclusion criteria **(Table 1)**. In cases where full texts were unavailable, study abstracts were included. Potentially relevant articles identified in the initial screening underwent full-text screening by the same reviewers. The final selection of studies included in this review received verification and approval from all reviewers. Any screening conflicts that arose were resolve by a neutral fifth reviewer (DTZ).

**Table 1:** Eligibility criteria for a scoping review on abortion provision after *Dobbs v*. *Jackson*.

**The following articles were included:**
Any adult patient population (either general or specialized) based in the United States Includes the exposure of interest (i.e., *Dobbs v*. *Jackson* ruling) Evaluates the patient-centered outcomes of interest (i.e., access to abortion and reproductive health services, health outcomes related to physical and psychological wellbeing) Evaluates the provider-centered outcomes of interest (i.e., access to comprehensive abortion training, facilitators and barriers to providing abortion services) English language Studies with primary quantitative data (e.g., primary observational studies) Published after May 2, 2022 (when the U.S. Supreme Court’s draft of *Dobbs* became leaked)
**The following articles were excluded:**
Patient populations outside the United States Does not include the exposure of interest Does not include information on the patient- or provider-centered outcomes of interest No English language Studies with only qualitative data Studies with only secondary data (e.g., reviews, editorials, letters to the editor, etc.) Published before May 2, 2022

### 2.4 Data charting process and items

After the completion of full-text screening, one reviewer (DTZ) performed data extraction and all other co-authors verified the data. Relevant information was systematically collected and entered into a data extraction form with predefined endpoints, such as publication year, authors, study design, data collection period, data sources, methods and data analysis, and key outcomes and findings related to abortion and reproductive health services. The data extraction template comprised two sections, one for patient-oriented studies and another for studies involving medical trainees and healthcare providers. Preprints and abstracts retained after screening were updated with their final peer-reviewed versions if available.

### 2.5 Analysis, synthesis, and presentation of results

The final sample of studies underwent thematic analysis, including topics such as contraception (e.g., permanent contraception [PC], emergency contraception [EC], and other forms], the barriers and facilitators experienced by patients (and providers) with accessing (and providing) abortion care, clinical outcomes, and public attitudes pertinent to abortion and reproductive health services following the *Dobbs* ruling.

## 3. Results

### 3.1 Sample and article characteristics

**Fig 1:**
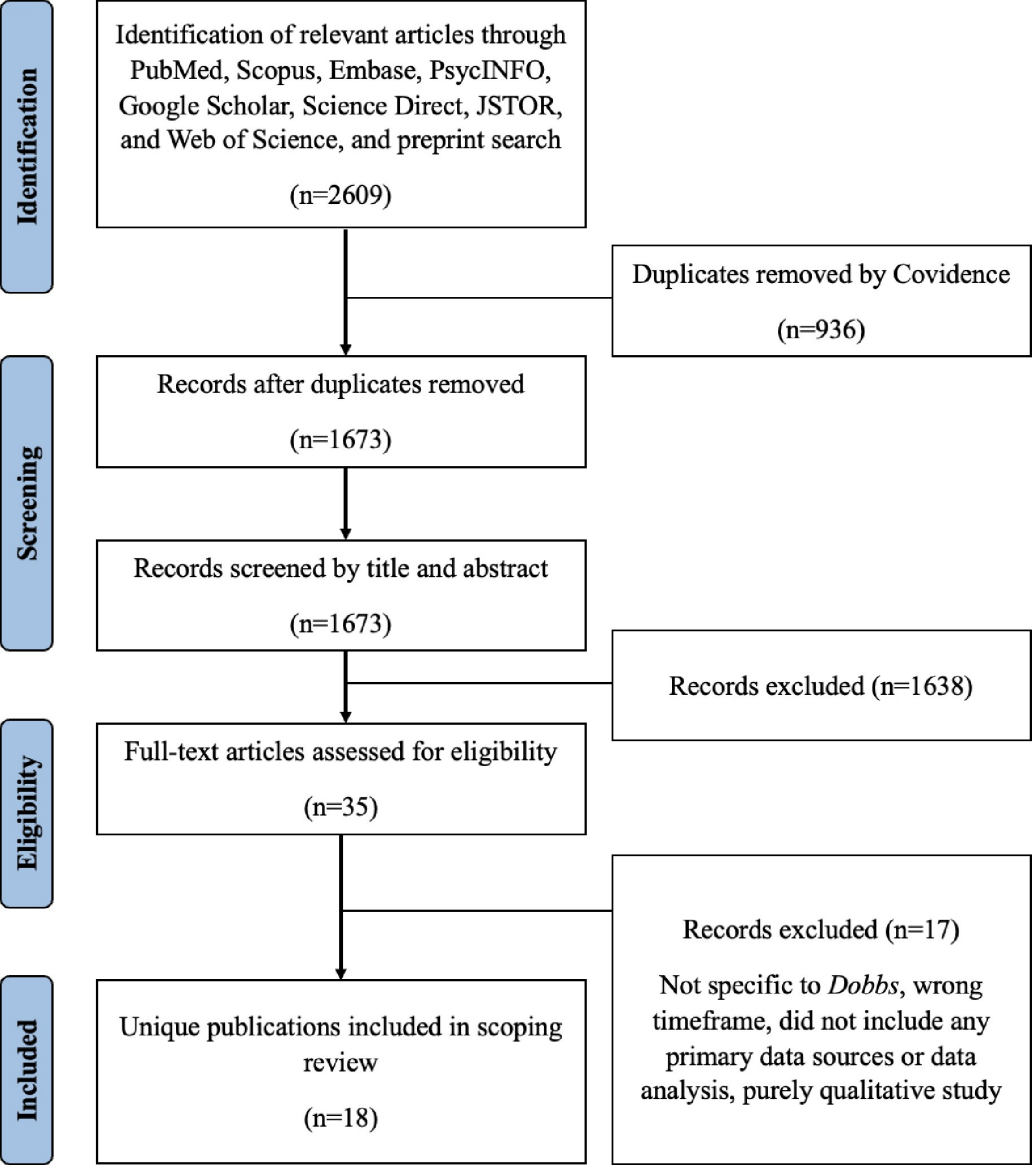
PRISMA flow diagram.

Our initial search yielded 2,609 articles. Automatic deduplication by Covidence removed 936 articles. Subsequently, title and abstract screening excluded 1,638 articles and full-text screening removed an additional 17 articles. Our final sample comprised 18 articles (12 full-text articles and 6 study abstracts), all subjected to data extraction. The screening process yielded a Cohen’s Kappa score of 0.82. An overview of our screening process is presented in **(Fig 1)**. The final sample consisted predominantly of cross-sectional (n = 6) [25, 27, 29, 31, 32, 34], modeling (n = 5) [23, 24, 26, 30, 35], and observational (n = 4) study designs [19–22], along with was one retrospective chart review [18], one NLP-based study [28], and one commentary [33] **(Table 2)**.

**Table 2:** Study characteristics and major findings for a scoping review on abortion provision after *Dobbs v*. *Jackson*.

Publication	Authors	Study Design	Population	Data Collection Period	Data Sources	Outcome(s)	Magnitude
***Patient-focused studies***							
Rising vasectomy volume following reversal of federal protections for abortion rights in the United States [18]	Bole et al.	Retrospective chart review (full text available)	Adults seeking vasectomies	July to August 2021 (pre-*Dobbs*); July to August 2022 (post-*Dobbs*)	Vasectomy procedural billing data	Vasectomy consultation requests post-*Dobbs*	+35.0%
						Vasectomy consultations post-*Dobbs*	+22.4%
						Monthly vasectomies completed post-*Dobbs*	+109.6%
Search trends signal increased vasectomy interest in states with sparsity of urologists after overrule of Roe vs. Wade [19]	Patel et al.	Observational (full text available)	No restrictions	March 25, 2022 to June 29, 2022	Google Trends	Mean RSV^a^ for “vasectomy” searches post-*Dobbs* in prohibited states	78.5%
						Mean RSV^a^ for “vasectomy” searches post-*Dobbs* in legal states	64.2%
The unprecedented increase in Google searches for “vasectomy” after the reversal of Roe vs. Wade [20]	Sellke et al.	Observational (full text available)	No restrictions	July 2017 to July 2022	Google Trends	Mean RSV^a^ difference for “vasectomy” searches two weeks post-*Dobbs* (versus two weeks pre-*Dobbs*)	+30.1%
						RSV^a^ for “vasectomy” searches on day of *Dobbs* passing	100%
						RSV^a^ for “vasectomy” searches on day of leaked *Dobbs* draft	60%
						RSV^a^ for “vasectomy” searches on day of Alabama House Bill 314	55%
The interest in permanent contraception peaked following the leaked Supreme Court majority opinion of Roe vs. Wade: a cross-sectional Google Trends analysis [21]	Ghomeshi et al.	Observational (full text available)	No restrictions	April 25, 2022 to May 8, 2022	Google Trends	Mean SVI^a^ difference for “vasectomy” searches one week after leaked *Dobbs* draft (versus one week pre-*Dobbs*)	+121%
						Mean SVI^a^ difference for “tubal ligation” searches one week after leaked *Dobbs* draft (versus one week pre-*Dobbs*)	+70%
Looking for a silver lining to the dark cloud: a Google Trends analysis of contraceptive interest in the United States post Roe vs. Wade verdict [22]	Datta et al.	Observational (full text available)	No restrictions	April 6, 2022 to July 5, 2022	Google Trends	Mean SVI^a^ difference for “vasectomy” searches post-*Dobbs* (versus June 23, 2022)	+614%
						Mean SVI^a^ difference for “tubal ligation” searches post-*Dobbs* (versus June 23, 2022)	+489%
						Mean SVI^a^ difference for “IUD” searches post-*Dobbs* (versus June 23, 2022)	+80%
						Mean SVI^a^ difference for “birth control pill” searches post-*Dobbs* (versus June 23, 2022)	+75%
						Mean SVI^a^ difference for “condom” searches post-*Dobbs* (versus June 23, 2022)	+57%
						Mean SVI^a^ difference for “morning after pill” searches post-*Dobbs* (versus June 23, 2022)	+700%
Impact of banning emergency contraception in states with abortion bans: a cost-effectiveness analysis [23]	Dzubay et al.	Modeling (abstract only)	Theoretical cohort of people capable of pregnancy in states with abortion bans under a hypothetical EC ban	—	—	Abortions post-*Dobbs* under theoretical EC ban (versus no EC ban)	+41,052 cases (+78.36%)
						Miscarriages post-*Dobbs* under theoretical EC ban (versus no EC ban)	+11,168 cases (+78.35%)
						Pre-eclampsia cases post-*Dobbs* under theoretical EC ban (versus no EC ban)	+1,611 cases (+77.98%)
						Maternal deaths post-*Dobbs* under theoretical EC ban (versus no EC ban)	+4 cases (+50.00%)
						Preterm births post-*Dobbs* under theoretical EC ban (versus no EC ban)	+3,839 cases (+78.36%)
						Neonatal deaths post-*Dobbs* under theoretical EC ban (versus no EC ban)	+83 cases (+79.05%)
						Number of neurodevelopmental cases post-*Dobbs* under theoretical EC ban (versus no EC ban)	+34 cases (+89.47%)
						Healthcare costs under theoretical EC ban (versus no EC ban)	+$541,716,923 (+72.50%)
						QALYs under theoretical EC ban (versus no EC ban)	–13,643 QALYs (–0.03%)
Estimated travel time and spatial access to abortion facilities in the US before and after the Dobbs v Jackson Women’s Health decision [24]	Rader et al.	Modeling (full text available)	Females of reproductive age living in the U.S.	January to December 2021 (pre-*Dobbs*); September 2022 (modeled post-*Dobbs* period, assuming complete abortion facility closures in states with total or 6-week bans)	Advancing New Standards in Reproductive Health database, 2020 American Community Survey	Median surface travel time to abortion facilities post-*Dobbs* (versus pre-*Dobbs*)	+6.1 minutes
						Mean surface travel time to abortion facilities post-*Dobbs* (versus pre-*Dobbs*)	+72.6 minutes
						Proportion living more than 60 minutes from an abortion facility post-*Dobbs* (versus pre-*Dobbs*)	+18.7%
Requests for self-managed medication abortion provided using online telemedicine in 30 US states before and after the Dobbs v Jackson Women’s Health Organization decision [25]	Aiken et al.	Cross-sectional (full text available)	Individuals requesting self-managed medications abortions from Aid Access	September 1, 2021 to May 1, 2022 (baseline); May 2, 2022 to June 23, 2022 (after leaked *Dobbs* draft); June 24, 2022 to August 31, 2022 (post-*Dobbs* passing)	Aid Access	Mean daily requests for self-managed medication abortions after leaked *Dobbs* draft	+54.5 daily requests
						Mean daily requests for self-managed medication abortions post-*Dobbs*	+131.1 daily requests
Predicted changes in travel distance for abortion among counties with low rates of effective contraceptive use following Dobbs v Jackson [26]	Rodriguez et al.	Modeling (full text available)	Reproductive age Medicaid recipients (ages 15–44 years)	—	Medicaid Transformed Medicaid Statistical Information System Analytic Files	Proportion of participants in counties with low contraceptive use and restricted abortion access post-*Dobbs* (versus pre-*Dobbs*)	+36%
Characteristics of people obtaining abortions in states likely to ban it: findings from a 2021–2022 national study [27]	Jones & Chiu	Cross-sectional (abstract only)	Individuals obtaining abortions in a random sample of abortion facilities	June 2021 to June 2022	Survey	Proportion of non-Hispanic Black individuals within abortion-hostile states (versus abortion-safe states)	+15%
						Proportion of non-Hispanic White individuals within abortion-hostile states (versus abortion-safe states)	+7%
						Proportion currently receiving medication abortions in abortion-hostile states (versus abortion-safe states)	+11%
						Proportion traveling out-of-state to an abortion-hostile state (versus abortion-safe states)	+2.1%
						Proportion paying out of pocket for an abortion	87%
						Proportion facing financial barriers for an abortion	56%
Examination of the public’s reaction on Twitter to the over-turning of Roe v Wade and abortion bans [28]	Mane et al.	NLP (full text available)	1% random sample of publicly available tweets based on keywords related to *Roe v*. *Wade* and abortion	May 1, 2021 to July 15, 2021 (pre-*Dobbs*); May 1, 2022 to July 15, 2022 (post-*Dobbs*)	Twitter API for Academic Research	Proportion of “negative” tweets on *Roe v*. Wade and abortion post-*Dobbs* (versus pre-*Dobbs*)	+0.17%
						Proportion of “neutral” tweets on *Roe v*. Wade and abortion post-*Dobbs* (versus pre-*Dobbs*)	+2.55%
						Proportion of “positive” tweets on *Roe v*. Wade and abortion post-*Dobbs* (versus pre-*Dobbs*)	–4.71%
Impact on access to methotrexate in the post-Roe era [29]	Wipfler et al.	Cross-sectional (abstract only)	Adults participating in FORWARD	—	FORWARD Survey (the National Databank for Rheumatic Diseases)	Proportion that experienced new challenges with methotrexate access post-*Dobbs*	1.25%
Abortion restriction impact on burden of neonatal single ventricle congenital heart disease: a decision-analytic model [30]	Miller et al.	Modeling (abstract only)	Theoretical cohort of neonates under various policy scenarios	—	—	Incidence of SVCD per 100,000 live births under a complete ban post-*Dobbs* (versus pre-*Dobbs*)	+10.8%
						Incidence of SVCD per 100,000 live births under a partial ban beyond 13 weeks post-*Dobbs* (versus pre-*Dobbs*)	+10.0%
						Incidence of SVCD per 100,000 live births under a partial ban beyond 20 weeks post-*Dobbs* (versus pre-*Dobbs*)	+7.7%
						Incidence of SVCD-related heart surgery per 100,000 live births under a complete ban post-*Dobbs* (versus pre-*Dobbs*)	+9.4%
						Incidence of SVCD-related heart surgery per 100,000 live births under a partial ban beyond 13 weeks post-*Dobbs* (versus pre-*Dobbs*)	+8.8%
						Incidence of SVCD-related heart surgery per 100,000 live births under a partial ban beyond 20 weeks post-*Dobbs* (versus pre-*Dobbs*)	+6.9%
						Incidence of SVCD-related death per 100,000 live births under a complete ban post-*Dobbs* (versus pre-*Dobbs*)	+3.1%
						Incidence of SVCD-related death per 100,000 live births under a partial ban beyond 13 weeks post-*Dobbs* (versus pre-*Dobbs*)	+2.8%
						Incidence of SVCD-related death per 100,000 live births under a partial ban beyond 20 weeks post-*Dobbs* (versus pre-*Dobbs*)	+0.3%
***Provider-focused studies***							
Fellow perspectives of abortion-related training in maternal-fetal medicine fellowship: regional differences in a post-Roe world [31]	Cheng et al.	Cross-sectional (abstract only)	Maternal-fetal medicine fellows	June 2022	Survey	Proportion of fellows affiliated with CFP in abortion-safe states (versus abortion-hostile states)	+22.2%
						Proportion of fellows affiliated with FI centers in abortion-safe states (versus abortion-hostile states)	–15.3%
						Proportion of fellows rating their aculty’s pro-abortion legislative advocacy as an important factor in abortion-safe states (versus abortion-hostile states)	+19.5%
						Proportion participating in pro-abortion advocacy in abortion-safe states (versus abortion-hostile states)	+17.6%
Trainee opinions regarding the effect of the Dobbs v. Jackson women’s health organization Supreme Court decision on obstetrics and gynecology training [32]	Meriwether et al.	Cross-sectional (abstract only)	OB-GYN residents	October 31, 2022	Survey	Proportion of residents believing they will be prohibited from providing standard of care post-*Dobbs* for the following services:	
						Early pregnancy loss	4.98%
						Assisted reproductive technologies involving embryos	9.2%
						Induced abortion in first trimester (any indication)	35.1%
						Induced abortion in second trimester (any indication)	38.5%
						Proportion of residents concerned about receiving charges post-*Dobbs* for providing the following services:	
						Early pregnancy loss	31.7%
						Assisted reproductive technologies involving embryos	38.2%
						Induced abortion in first trimester (any indication)	55.6%
						Induced abortion in second trimester (any indication)	63.0%
						Management of abortion complications	48.36%
Projected implications of overturning Roe v Wade on abortion training in U.S. obstetrics and gynecology residency programs [33]	Vinekar et al.	Commentary (full text available)	Residents in accredited U.S. OB-GYN residency programs	—	American Medical Association database of OB-GYN residency programs in the U.S. and the Guttmacher Institute	Proportion of OB-GYN residents within states certain to ban abortion	38.4%
						Proportion of OB-GYN residency programs within states certain to ban abortion	38.8%
						Proportion of OB-GYN residents within states likely to ban abortion	5.5%
						Proportion of OB-GYN residency programs within states likely to ban abortion	5.9%
Forensic nurses’ understanding of emergency contraception mechanisms: implications for access to emergency contraception [34]	Downing et al.	Cross-sectional (full text available)	Sexual assault nurse examiners within the International Association of Forensic Nurses	September 28, 2022 to October 1, 2022	Survey	Proportion believing their EC prescribing will increase post-*Dobbs*	6.94%
						Proportion believing their EC prescribing will decrease post-*Dobbs*	0.58%
						Proportion believing their EC prescribing will not change post-*Dobbs*	79.77%
						Proportion unsure if their EC prescribing will change post-*Dobbs*	12.72%
Presence and absence: crisis pregnancy centers and abortion facilities in the contemporary reproductive justice landscape [35]	Thomsen et al.	Modeling (full text available)	—	—	Advancing New Standards In Reproductive Health database, Reproaction Fake Clinic database, IPUMS National Historical GIS project, National Center for Health Statistics	Proportion living in areas with CPCs closer than abortion facilities post-*Dobbs* (versus pre-*Dobbs*)	+26.5%
						Proportion living in areas with abortion facilities closer than CPCs post-*Dobbs* (versus pre-*Dobbs*)	–0.1%
						Proportion living in areas with CPCs and abortion facilities equidistant post-*Dobbs* (versus pre-*Dobbs*)	–26.4%
						Proportion living in areas within 30 minutes of an abortion facility post-*Dobbs* (versus pre-*Dobbs*)	–24.7%
						Proportion living in areas within 60 minutes of an abortion facility post-*Dobbs* (versus pre-*Dobbs*)	–9.6%
						Proportion living in areas more than 120 minutes from an abortion facility post-*Dobbs* (versus pre-*Dobbs*)	–38.9%

**Note:** RSV, relative search volume; SVI, Search volume index; EC, emergency contraception; CFP, complex family planning; FI, fertility and infertility; SVCD, single ventricle congenital heart disease; OB-GYN, obstetrics and gynecology; QALYs, quality-adjusted life years; NLP, natural language processing; EC, emergency contraception; CPC, crisis pregnancy center.

^a^RSV and SVI measures the search popularity of a given topic, relative to its peak popularity, on a scale of 1–100

### 3.2 Contraception

Seven studies (38.9%) discussed contraception **(Table 2)** [18–23, 34]. Following the *Dobbs* ruling, these studies consistently revealed an increased demand for PCs. Google searches for vasectomies dramatically increased after *Dobbs* passed on June 24, 2022, and to a smaller extent after the U.S. Supreme Court’s draft of *Dobbs v*. *Jackson* leaked on May 2, 2022 [19–22]. Oklahoma, South Dakota, Idaho, New Mexico, and Hawaii exhibited the highest ‘vasectomy’ search rates [22]. This rise in Google searches aligns with an actual increase of vasectomy requests, consultations, and procedures, identified using vasectomy billing data [18]. Notably, younger men, particularly those below 30 years and without children, were more likely to seek vasectomy consultations after *Dobbs* [18]. Interestingly, the demand for vasectomies exhibited an inversely correlation to the ratio of urologists to adult men in states, indicating a potential strain on the urological workforce and increased delays [19]. Similarly, there was a rise in Google searches for ‘tubal ligation’ after *Dobbs*, although less pronounced than for vasectomies [21, 22]. The Northern and Southwestern U.S. regions experienced the greatest surge in Google searches for vasectomies, while the Midwestern regions experienced the greatest surge in tubal ligation searches [21].

Similarly, the demand for various ECs surged following *Dobbs* **(Table 2)**. Google searches for ‘morning after pill’ rose by approximately eight-fold after *Dobbs*, with the most significant uptick in Idaho, District of Columbia (DC), South Dakota, Oklahoma, and North Dakota [22]. Further, a modeling study projected that that maintaining access to ECs after *Dobbs* for a theoretical cohort of 750,000 patients capable of pregnancy was associated with a reduction in 41,052 abortions, 11,168 miscarriages, 1,611 cases of preeclampsia, 3,839 preterm births, 4 maternal deaths, 83 neonatal deaths, and 34 neurodevelopmental delays, illustrating the substantial clinical and public health advantages of ensuring equitable access to ECs. Additionally, it would be associated with an additional 13,634 quality-adjusted life-years (QALYs) and US$541,716,923 in healthcare expenditure savings [23]. Furthermore, one study analyzed forensics nurses’ attitudes towards the *Dobbs* ruling and found that, while a minority believed that EC prescribing would decrease (0.58%) or increase (6.94%), the majority believed that *Dobbs* would not affect the prescribing of ECs (79.77%) [34]. Concerns about current or legal restrictions surrounding EC prescribing after Dobbs, including fear of prosecution, were cited by several nurses [34].

Additionally, in the aftermath of *Dobbs*, there was an evidence surge in demand for various other contraceptives **(Table 2)**. Google searches also increased for ‘IUD’, ‘birth control pill’, and ‘condom’after *Dobbs*, although less pronounced than for PCs and ECs.^22^ The upswing of Google searches for ‘IUD’ was highest among states such as Utah, DC, Montana, Colorado, Minnesota, while Google searches for ‘condom’ were highest among states such as Delaware, New York, New Jersey, Connecticut, Mississippi [20]. The authors attributed the comparatively modest rise in Google searches for ‘birth control pills’ to several potential factors, including lack of awareness, concerns regarding efficacy or potential side effects, financial barriers, or lack of convenience [22].

### 3.3 Medications and medical conditions

Three studies (16.7%) discussed the impact of *Dobbs* on medications other than contraceptives **(Table 2)** [25, 29, 30]. One study found that requests for self-managed abortion medications via Aid Access, a telemedicine nonprofit organization that enables individuals to order abortion medications via mail [36], rose from 82.6 to 137.1 mean daily requests after the leaked draft of *Dobbs* on May 2, 2022, followed by a further increase to 213.7 mean daily requests after *Dobbs* officially passed [25]. The requests rose in every U.S. state, although states that implemented total bans experienced the largest increase [25]. The number of requesters citing “current abortion restrictions” as a primary reason increased from 31.4% to 62.4% after *Dobbs*, which tended to be most pronounced in abortion-hostile states but were also prevalent in states in which state laws governing abortion did not immediately change after *Dobbs* [25]. Patients seeking other medications capable of inducing abortion after *Dobbs* also faced difficulties, notably, with regards to methotrexate. A study revealed that approximately 1 in 17 people experienced unexpected barriers to accessing methotrexate after *Dobbs*, of which 21.7% were directly related to *Dobbs* (e.g., prescription delays or refusals citing pregnancy risks or concerns related to abortion) [29]. Finally, one study revealed the significant impact of Dobbs on the incidence of births complicated by life-limiting fetal anomalies, notably neonatal single ventricle congenital heart disease (SVCD) [30]. The authors found that, under less restrictive abortion restrictions (e.g., ban on abortions beyond 20 weeks), the incidence of SVCD per 100,000 live births and SVCD-related surgeries is comparatively less than under more restrictive partial bans (e.g., ban on abortions beyond 13 weeks) or complete abortion bans [30].

### 3.4 Travel as a barrier to abortion access

Four studies (22.2%) focused on travel-related barriers to accessing abortion clinics and other reproductive health services **(Table 2)** [24, 26, 27, 35]. These studies consistently found that state laws under *Dobbs* would compound existing such barriers. According to a modeling study, the percentage of women facing restricted access to both contraception and abortion facilities was projected to increase from 11% (pre-*Dobbs*) to 46% (post-*Dobbs*), affecting approximately 1.6 million women across 34 U.S. states [26]. Similarly, another modeling study found that, between January 2021 to September 2022, the mean surface travel time (e.g., by car or public transport) to abortion facilities increased from 27.8 minutes (pre-*Dobbs*) to 100.4 minutes (post-*Dobbs*) [24]. As such, the percentage of reproductive-age women living in a census tract more than 60 minutes from an abortion facility increased from 14.6% (pre-*Dobbs*) to 33.3% (post-*Dobbs*) [24].

Notably, the impact of travel-related barriers on accessing abortion care was disproportionately felt by racial minority populations. Census tracts located more than 60 minutes from an abortion facility predominantly comprised residents of racial and ethnic minorities with a lower mean household income and lacking health insurance, a high school diploma, or internet access [24]. Further, the increase in prevalence of reproductive-age females living in a census tract more than 60 minutes from an abortion facility among Black (25.6% increase), Hispanic (21.7% increase), and American Indian or Alaskan Native (20.4% increase) populations surpassed non-Hispanic White reproductive-age females (18.0% increase) [24]. In contrast, this magnitude of this increased prevalence was smaller for Asian (14.1% increase) and Native Hawaiian or Pacific Islander (11.8% increase) reproductive-age females [24]. Racial and ethnic disparities were also evident in the context of the relative proximity of crisis pregnancy centers (CPCs) to abortion facilities. A modeling study predicted an increase in the CPCs-to-abortion facility ratio from approximately 3:1 to 5:1 following expected service restrictions or closures of abortion facilities post-*Dobbs* [35]. This increased ratio affects Hispanic (29.6% increase) more than White patients (24.4% increase), followed by Black (23.6% increase), Native American or Alaskan Native (20.6% increase), and Asian or Pacific Islander (19.2% increase) patients [35]. Finally, a survey highlighted that individuals living in abortion-restricted states were more likely to experience travel-related barriers, rely on financial assistance, pay out-of-pocket for abortion care, and encounter financial obstacles related to abortion- or reproductive health-related services [27].

### 3.5 Public attitudes

Only one study (5.6%) discussed public attitudes related to abortion care and family planning in the post-*Dobbs* landscaping **(Table 2)** [28]. Through sentiment analysis of tweets (social media posts) on the platform Twitter, the authors employed a machine learning algorithm to dichotomously classify tweets as either “positive” (supportive) or “negative” (unsupportive) concerning various topics related to abortion and reproductive health [28]. Their findings revealed a growing polarization after *Dobbs*, driven by a small (0.17%) increase in the percentage of overall negative tweets towards abortion and *Roe v*. *Wade*, accompanied by a modest (4.71%) decrease in positive tweets [28]. Such changes were most pronounced for tweets concerning “Roe v. Wade”, with a 10.8% increase and 5.63% decrease in negative and positive tweets, respectively; followed by tweets concerning “family planning”, with a 5.35% increase and 3.28% decrease in negative and positive tweets, respectively [28]. “Pro-life” tweets typically centered around personal religious belief or support for conservative policies underpinning “pro-life” movements [28]. In contrast, “pro-choice” (pro-abortion) tweets typically expressed anger and dismay with the *Dobbs* decision, highlighted the need for preserving access to abortion care, demonstrated fear over potential loss of access to contraception after *Dobbs*, and perceived *Dobbs* as a violation of fundamental human rights for people capable of pregnancy [28].

### 3.6 Medical residency and fellowship programs

Three studies (16.7%) described the impact of the *Dobbs* ruling on the training and clinical practice of medical trainees such as residents and fellows **(Table 2)** [31–33]. In a study examining 286 OB-GYN residency training programs across the U.S., it was found that 5.9% and 38.8% of programs are located in states either likely or certain to ban comprehensive training related to abortion care provision after *Dobbs*, respectively [33]. This translates to 43.9% of OB-GYN residents, totaling 2,638 individuals, training in programs located in states likely or certain to ban abortion after *Dobbs* [33].

Further, substantial concerns arose regarding the potential impact of *Dobbs* on restricting healthcare providers’ scope of practice. Notably, 35.1% and 38.5% of U.S. OB-GYN residents believed that they would have to cease providing standard-of-care for induced abortion during the first and second trimester, respectively, post-*Dobbs* [32]. Further, 55.6% and 63.0% expressed fear of facing charges for providing the standard-of-care for induced abortion during the first and second trimester, respectively, post-*Dobbs* [32]. Notably, 48.36% expressed fear of facing charges for clinical management of abortion complications following *Dobbs* [32].

Finally, another study found that maternal-fetal medicine (MFM) fellowship programs in abortion-hostile states were more likely to be associated with fertility and infertility (FI) centers, while MFM fellowships in abortion-friendly states are more likely to be associated with complex family planning (CFP) fellowship [31]. Moreover, MFM fellows in abortion-friendly states are more likely to be female, participate in pro-abortion advocacy, and placed a higher emphasis on abortion-related training in their fellowship training [31].

## 4. Discussion

This first scoping review is the first to examine the public health and clinical implications of the *Dobbs v*. *Jackson* on patients, medical trainees (e.g., residents), and healthcare providers, to the best of our knowledge. Our preliminary findings contribute to understanding the multifaceted impact of the *Dobbs* ruling on access to, and the provision of, abortion care and reproductive health services. This encompasses diverse aspects, including various contraception methods, health service accessibility, social and structural barriers (e.g., travel- and cost-related barriers to access), public attitudes, and medical training programs.

Our findings offer valuable insights into the significant deleterious effects resulting from restricted access to legal abortion care in numerous U.S. states post-*Dobbs*. This underscores the urgent need for legal, public health, and clinical interventions focused on implementing equity-centered policies designed to enhance accessibility to contraception, abortion care, and reproductive health services following *Dobbs*. Prohibiting access to safe and legal abortions poses a significant risk to maternal morbidity and mortality, leading to enduring physical, psychological, and socioeconomic consequences for both the child and the mother [37, 38]. The World Health Organization (WHO) corroborates the live-saving potential of decriminalizing abortion and ensuring equitable access, both of which are crucial aspects of its 2022 guidelines that aims to prevent over 25 million unsafe abortions annually among individuals capable of pregnancy [37]. Therein lies a social and public health imperative to advocate for reproductive health equity and abortion care decriminalization and access. Achieving these goals will require strengthened partnerships between clinical and community-based stakeholders to allocate resources such as contraception, transportation funding to abortion-friendly neighboring states, mental health support, and other essential services in the aftermath of *Dobbs* [37].

Several studies [18–23, 34] in this review detailed the impact of *Dobbs* on demand for, and access to, PCs (e.g., vasectomies and tubal ligation), ECs, and various other contraceptives (e.g., IUD, birth control pill, condoms, etc.). The surge in Google searches for contraception, along with increased consultations and vasectomies performed (as identified by billing data) [18], demonstrates heightened interest and demand for contraception post-*Dobbs*. Moving forwards, public health interventions should prioritize expanding the affordable distribution of contraception supply, particularly within historically marginalized and medically underserved communities that are more likely to reside in contraceptive deserts and encounter structural (e.g., financial) barriers to contraception [39–41]. A greater demand for contraception following *Dobbs* may potentially contribute to shortages in contraception, for which the greatest burden will likely be felt by vulnerable populations, although more research is needed. Similarly, studies consistently demonstrated that demand for contraception after *Dobbs* tended to be more concentrated in U.S. states with more restrictive abortion laws [19, 21, 22], a concerning trend given the disproportionately higher density of contraceptive deserts within such states [39–41]. Of further concern, one study found that patients experienced novel barriers to other medications such as methotrexate after *Dobbs* due to providers’ hesitancy or refusal to prescribe them since they are capable of inducing abortion [29]. This potentially foreshadows the encroachment of the *Dobbs* ruling on other fields such as oncology or rheumatology, calling for cross-specialty and collective efforts within the healthcare system to systematically address these emerging challenges.

Moreover, travel and financial concerns emerged as frequent barriers to accessing abortion care in the post-*Dobbs* landscape [24, 26, 27, 35]. Such barriers have been extensively documented in the existing literature, complicating health service accessibility due to the geographical inaccessibility of abortion facilities [10], legal restrictions necessitating cross-state travel to access reproductive health services [42], time constraints [10], cost burdens involving time off work and childcare services [43], emotional distress [44], among other mechanisms [45]. Notably, these barriers are most pronounced for medically underserved populations—including Black, Latinx, uninsured, undocumented, and low-income individuals—posing particular concern as these groups also exhibit the highest rates of maternal and pregnancy-related mortality [46]. Preliminary evidence indicates that *Dobbs* is expected to expand and compound these barriers and disparities [24–27], emphasizing the urgency for cross-sectoral, multi-level partnerships to address these social and structural barriers to abortion care post-*Dobbs* [41].

Finally, several studies [31–33] consistently discussed concerns among medical trainees (e.g., medical residents and fellows) about the impact of *Dobbs* on their scope of practice, especially given the large proportion of OB-GYN-related residency and training programs situated in abortion-hostile U.S. states. Medical residents frequently expressed concerns about being denied their ability to legally provide standard-of-care services for patients in need of abortions and similar reproductive health services, coupled with a fear of legal prosecution, sanctions, and charges for offering these services amidst the new restrictions under *Dobbs* [32]. It is evident that *Dobbs* has already severely curtailed medical trainees’ abilities to receive training for, and healthcare providers’ abilities to provide, the full spectrum of abortion services in abortion-hostile states. Further advocacy from regional and national healthcare is necessary to enhance training and clinical practice related to abortion care in the aftermath of *Dobbs*. This could include promoting cross-institutional partnerships, securing funding, and establishing transportation networks, thereby allowing medical residents in abortion-hostile states to access abortion training from neighbouring abortion-friendly states. Additionally, improving current approaches to data sharing and knowledge translation may help clinicians navigate the rapidly evolving landscape of diverse hospital policies and state laws governing abortion care. This may include developing effective strategies for accessing up-to-date information on legal developments and institutional guidelines, in addition to resources and support networks to promote clinicians’ wellbeing, prevent moral distress, and mitigate burnout.

### 4.1 Limitations

There are several limitations to our study. Firstly, our scoping review captured articles published only nine months after the passing of *Dobbs v*. *Jackson*, aiming to conduct a preliminary exploratory analysis to guide hypothesis generation in future studies. Consequently, our data sources were limited and our findings are not intended to be representative of the general U.S. population. In particular, more research is warranted to comprehensively describe the implications of *Dobbs* on patients and healthcare providers at a national level with a longer follow up period. It should also be noted that one-third of our articles reviewed were only available in their abstract form, indicating that our findings are preliminary and may be subject to change once the full-text articles become available. Secondly, we did not attempt to quantitatively synthesize the percentages and rates described in **(Table 2)**, and a systematic review and meta-analysis might be warranted in the future. Thirdly, our screening process involved four reviewers, which may have introduced inconsistencies. Although we achieved a relatively high kappa score (0.82) and attempted to mitigate these inconsistencies by training all reviewers (e.g., screening a subset of articles together to help achieve a more consistent reasoning process), this may have still affected the reliability of our screening and findings. Fourthly, only 16.7% (n = 3/18) studies in this review were related to the implications of the *Dobbs* ruling on the training and clinical practice of medical residents and fellows. There is a significant need for further epidemiological studies to quantitatively evaluated the impact of *Dobbs* on medical training programs and clinical practice in OB-GYN and related fields, as well as in other healthcare professions. Finally, we excluded qualitative studies in our current review to concentrate on quantitative findings from observational studies. However, a future review that includes qualitative studies and identifies key themes related to patients’ and providers’ lived experiences after Dobbs may be appropriate. Qualitative studies are crucial to understanding the nuanced and subjective experiences of healthcare professionals in the aftermath of *Dobbs*, providing valuable context to complement the findings from quantitative and observational studies highlighted in the present study.

## 5. Conclusion

*Dobbs v*. *Jackson* has imposed significant deleterious consequences to patients’ access to abortion care in the U.S., and hindered healthcare providers’ capacity to deliver the complete spectrum of abortion care and reproductive health services. The public health consequences of this ruling are undeniable, further stretching existing social and structural vulnerabilities among populations already experiencing significant disparities in maternal mortality and pregnancy-related outcomes. Consequently, urgent actions and research are needed from multiple spheres of action—healthcare providers, policymakers, legislators, public health agencies, and the public—to further map and address the consequences of *Dobbs* on the healthcare system and advocate for reproductive health equity in the evolving post-*Dobbs* landscape.

## Supporting information

S1 Table Preferred Reporting Items for Systematic reviews and Meta-Analyses extension for Scoping Reviews (PRISMA-ScR) checklist. (DOCX)

S2 Table Systematic review search strategy (restricted between 2022–2023). (DOCX)
