# Supplementary material for: Public health and clinical implications of Dobbs v. Jackson for patients and healthcare providers: A scoping review
Source: PLoS One. 2024 Mar 29;19(3):e0288947. doi: 10.1371/journal.pone.0288947 (PMC10980209; doi:10.1371/journal.pone.0288947)
Supplement: S2 Table — (DOCX) [file pone.0288947.s002.docx]

**Table S2.** Systematic review search strategy (restricted between 2022-2023)

| **Database** | **Search terms*** | **Number of results** |
| --- | --- | --- |
| PubMed | (abortion*[tiab]) OR (pregnan*[tiab] AND terminat*[tiab]) OR (unintended AND pregnan*[tiab]) OR (abortifacient*[tiab]) OR (mifepristone[tiab]) OR (mifeprex[tiab]) OR (misoprostol[tiab]) OR (cytotec[tiab]) AND (dobbs[tiab]) OR (roe[tiab]) Filters: Abstract, from 2022 - 2023 | 367 |
| Scopus | (TITLE-ABS-KEY (abortion*) OR  TITLE-ABS-KEY (pregnancy AND terminat*)  OR TITLE-ABS-KEY (unintended AND pregnanc*) OR TITLE-ABS-KEY (abortifacient*) OR TITLE-ABS-KEY (mifepristone) OR TITLE-ABS-KEY (mifeprex) OR TITLE-ABS-KEY (misoprostol) OR TITLE-ABS-KEY (cytotec) AND TITLE-ABS-KEY (dobbs OR roe)) AND (LIMIT-TO (PUBYEAR, 2023) OR LIMIT-TO (PUBYEAR, 2022)) | 264 |
| Embase | ((abortion* or (pregnancy and terminat*) or (unintended and pregnanc*) or abortifacient* or mifepristone* or mifeprex or misoprostol or cytotec) and (dobbs or roe)).mp. [mp=title, abstract, heading word, drug trade name, original title, device manufacturer, drug manufacturer, device trade name, keyword heading word, floating subheading word, candidate term word]  limit 1 to yr="2022 - 2023" | 236 |
| PsycINFO | ((abortion* or (pregnancy and terminat*) or (unintended and pregnanc*) or abortifacient* or mifepristone* or mifeprex or misoprostol or cytotec) and (dobbs or roe)).mp. [mp=title, abstract, heading word, table of contents, key concepts, original title, tests & measures, mesh word]  limit 1 to yr="2022 - 2023" | 11 |
| Google Scholar | (dobbs OR roe) AND study AND abortion OR "pregnancy termination" OR "termination of pregnancy" OR "unintended pregnancy" OR abortifacient OR mifepristone OR mifeprex OR misoprostol OR cytotec -legal -editorial -viewpoint -opinion | 322 |
| Science Direct | Dobbs OR Roe AND abortion  Restricted to 2022 (101), 2023 (63), Research Articles (110), Book Chapters (6), Conference Abstracts (48), and Other (16) | 450 |
| JSTOR | abortion OR termination of pregnacy OR mifepristone OR misoprostol OR Dobbs OR Roe  Restricted to 2022-2023, Research Articles, Research Reports, and Miscellaneous | 316 |
| Web of Science | abortion (Topic) AND (Dobbs OR Roe) (Topic)  Restricted to Publication Years: 2023 or 2022, Document Types: Article or Early Access | 113 |
| medRxiv | Dobbs Roe abortion abortifacient mifepristone misoprostol mifeprex cytotec (any)  Restricted to 2022-2023 | 33 |
| bioRxiv | Dobbs Roe abortion abortifacient mifepristone misoprostol mifeprex cytotec (any)  Restricted to 2022-2023 | 43 |
| Europe PMC | (abortion OR (pregnanc* AND terminat*) AND (Dobbs OR Roe)) AND (((SRC:MED OR SRC:PMC OR SRC:AGR OR SRC:CBA) NOT (PUB_TYPE:"Review")) OR SRC:PPR)  Restricted to 2022-2023 | 87 |
| Total number of articles | 2609 | |
| Total number of duplicates | 936 | |
| **Total after de-**  **depublication** | 1673 | |
